# Psychometric Evaluation of the Odia-Adapted Psychosocial Impact of Dental Aesthetics Questionnaire (PIDAQ): A Cross-Sectional Study Assessing the Psychosocial Impact of Malocclusion in Eastern Indian Adolescents

**DOI:** 10.7759/cureus.82658

**Published:** 2025-04-20

**Authors:** Vijeta Patri, Gaurav Patri, Nivedita Sahoo

**Affiliations:** 1 Department of Orthodontics, Hi-Tech Dental College and Hospital, Bhubaneswar, IND; 2 Department of Conservative Dentistry and Endodontics, Kalinga Institute of Dental Sciences, Kalinga Institute of Industrial Technology (KIIT) Deemed to be University, Bhubaneswar, IND; 3 Department of Orthodontics and Dentofacial Orthopedics, Kalinga Institute of Dental Sciences, Kalinga Institute of Industrial Technology (KIIT) Deemed to be University, Bhubaneswar, IND

**Keywords:** adolescent, aesthetics, malocclusion, orthodontic treatment need, pidaq (psychosocial impact of dental aesthetics questionnaire)

## Abstract

Introduction

The psychosocial impact of dental aesthetics significantly influences adolescents' emotional well-being, self-esteem, and social interactions. While the Psychosocial Impact of Dental Aesthetics Questionnaire (PIDAQ) has been validated across diverse populations, no version previously existed for the Odia-speaking adolescent population in Eastern India. This study aimed to translate, culturally adapt, and validate the PIDAQ for adolescents aged 10 to 13 years in Odisha.

Method

The original PIDAQ was translated and culturally adapted into Odia following forward and backward translation, expert panel review, and pilot testing. The finalized questionnaire was administered to 450 adolescents from urban and semi-urban schools in Odisha. Internal consistency was evaluated using Cronbach’s alpha, and test-retest reliability was assessed with intraclass correlation coefficients (ICC). Construct validity was determined through exploratory factor analysis, and discriminant validity was established by comparing scores across malocclusion severity categories using the Index of Orthodontic Treatment Need-Dental Health Component (IOTN-DHC) and Index of Orthodontic Treatment Need-Aesthetic Component (IOTN-AC) indices. Convergent validity was evaluated by correlating PIDAQ scores with IOTN-AC scores.

Results

The Odia version of the PIDAQ demonstrated good internal consistency, with Cronbach’s alpha values ranging from 0.72 to 0.78, and high test-retest reliability (ICC ranging from 0.71 to 0.84). Exploratory factor analysis confirmed a stable four-factor structure explaining 56.56% of the total variance. Discriminant validity was demonstrated through significant differences (p < 0.001) in PIDAQ scores across malocclusion severity groups. Moderate, statistically significant correlations between PIDAQ scores and IOTN-AC scores (ρ = 0.389) confirmed convergent validity.

Conclusion

The Odia-adapted PIDAQ is a reliable, valid, and culturally appropriate tool for assessing the psychosocial impact of dental aesthetics in adolescents, with significant potential for clinical use, public health research, and policy development.

## Introduction

The aesthetic appearance of teeth significantly influences adolescents’ psychosocial well-being, self-confidence, and social interactions, especially during a life stage marked by heightened self-awareness and peer influence [1-5]. While malocclusion is not life-threatening, it impacts facial aesthetics and is linked to psychological challenges such as anxiety, low self-esteem, and social withdrawal [6-8].

Clinical indices such as the Index of Orthodontic Treatment Need (IOTN), which includes the Dental Health Component (DHC) and the Aesthetic Component (AC), provide objective measures of malocclusion severity [5,6,9,10]. However, they often fail to capture patients’ subjective psychological burden [9,10]. To address this gap, the Psychosocial Impact of Dental Aesthetics Questionnaire (PIDAQ) was developed as a 23-item, self-administered instrument that evaluates four psychosocial domains: Dental Self-Confidence (DSC), Psychological Impact (PI), Social Impact (SI), and Aesthetic Concern (ACn) [1,2,6]. Compared to more generic oral health-related quality of life tools such as the Oral Health Impact Profile (OHIP-14), which measure broad functional limitations and discomfort, the PIDAQ provides a condition-specific, aesthetics-centered assessment [3,5]. This specificity is particularly critical in adolescence, a period characterized by heightened aesthetic sensitivity and vulnerability to peer evaluation [2,8,10]. The PIDAQ’s structured, aesthetics-oriented approach enables more accurate and clinically meaningful insights into the psychosocial dimensions of malocclusion [1]. It has demonstrated strong psychometric properties and has been validated across diverse cultural contexts, including Moroccan, Arabic, Chinese, Spanish, Persian, Croatian, Turkish, Malaysian, Albanian, and Indian (Hindi and Malayalam) populations [3-14].

In India, increasing aesthetic awareness has fueled orthodontic demand among adolescents, who are particularly vulnerable to psychological effects of perceived dental imperfections [2,8-10,15]. Despite prior validations in Hindi and Malayalam, no Odia version of the PIDAQ exists. Odia, an Indo-Aryan language widely spoken in Odisha and neighboring states, represents a large population with distinct linguistic and cultural characteristics [16]. The lack of a validated Odia PIDAQ limits comprehensive, patient-centered orthodontic care and overlooks critical psychosocial dimensions in treatment planning.

Furthermore, few studies have explored the relationship between PIDAQ scores and clinical indices like IOTN-DHC and IOTN-AC [3,5-7,10,13,17], a necessary step to ensure alignment between subjective needs and objective evaluations.

The primary objective of this research was to develop an Odia version of the PIDAQ through a systematic process of translation, cultural adaptation, and psychometric validation for adolescents aged 10-13 years residing in Bhubaneswar, Odisha. As part of the validation process, the study also explored the relationship between self-perceived dental aesthetics and clinically assessed malocclusion severity. The primary hypothesis focused on confirming the psychometric soundness, that is, specifically, the reliability and validity of the Odia-adapted PIDAQ. A secondary hypothesis, related to the assessment of discriminant and convergent validity, proposed that there would be no significant association between malocclusion severity and psychosocial impact as measured by the instrument.

## Materials and methods

Study design and ethical considerations

The purpose of this cross-sectional psychometric validation study was to translate and validate the English variant of the Psychosocial Impact of Dental Aesthetics Questionnaire (PIDAQ) [1,2] for adolescents in Odia. The Institutional Ethics Committee, Hi-Tech Medical College and Hospital, Bhubaneswar, provided ethical approval (HMCH/IEC/2024/136, dated February 26, 2024). School authorities and parents provided written informed consent, and all adolescent participants gave their assent.

Study setting and sampling procedure

The study was conducted among school-going adolescents aged 10 to 13 years in Bhubaneswar, Odisha. The city was stratified into three administrative zones, Bhubaneswar North, Central, and Ekamra. Two schools per zone (one government and one private) were randomly selected using the lottery method to ensure socioeconomic diversity. Cluster sampling was employed to enhance representativeness.

Sample size determination

The sample size was computed using the formula *n = (Z² × p × (1 - p)) / E²*, where *n* is the required sample size, *Z* = 1.96 corresponding to the Z-score for a 95% confidence level, *p* is the estimated prevalence, *E* is the margin of error set at 5%, and the calculation assumes 80% power. This yielded a minimum of 450 participants. To support psychometric validation, the yardstick by Plichta and Kelvin, that is, 10 participants per scale item, was also applied, indicating a minimum of 230 [18]. The final sample of 450 therefore exceeded both criteria, ensuring sufficient power for statistical and validation analyses. Cluster sampling was used to select schools across three zones in Bhubaneswar, with 75 students per school. Gender balance was maintained to support subgroup analysis and representativeness.

Inclusion and exclusion criteria

Inclusion criteria comprised Odia-speaking adolescents aged 10-13 years with no prior or ongoing orthodontic treatment. Exclusion criteria included craniofacial anomalies, extensive anterior caries affecting aesthetics, systemic conditions affecting oral health, and any ongoing dental or orthodontic intervention [9,10].

Clinical evaluation of treatment need

Orthodontic treatment needs were assessed utilizing both elements of the IOTN. For the DHC, clinical examinations were conducted following American Dental Association Type III standards using a mouth mirror and periodontal probe. Based on their DHC grades, participants were categorized into three groups: Group 1 included those with grades 1 and 2, indicating little or no treatment need; Group 2 comprised grade 3, reflecting borderline need; and Group 3 included grades 4 and 5, indicating definite need for orthodontic intervention. For the Aesthetic Component (AC), each participant was presented with a standardized 10-point photographic scale and asked to select the image that most closely resembled their own dental appearance. This dual assessment approach enabled the integration of objective clinical evaluation with subjective self-perception of dental aesthetics [10].

Translation and cross-cultural adaptation

The translation process followed international guidelines [19], including dual independent forward translations by bilingual experts, reconciliation by a multidisciplinary committee, and two independent back-translations. A pilot test was conducted with 30 adolescents to evaluate the clarity and cultural appropriateness of the finalized version. These participants were not included in the final validation analysis to avoid bias in reliability and validity assessment. Insights from their feedback led to slight refinements, ensuring alignment in semantic meaning, idiomatic usage, and conceptual understanding (see Appendices).

Reliability testing

Cronbach's alpha was used to determine internal consistency; values ≥0.70 were judged satisfactory. To assess test and retest reliability, a subset of 30 adolescents completed the questionnaire on two separate occasions, spaced two weeks apart. For test-retest reliability, a separate subgroup of 30 participants from the main validation sample (n = 450) completed the questionnaire again after a two-week interval. Only the responses from the main validation sample were included in the final psychometric analysis. Intraclass correlation coefficients (ICC) ≥0.75 indicated strong temporal stability [10].

Construct validation

The finalized Odia PIDAQ (see Appendices) was administered in a quiet setting with sufficient time for completion. Items were scored on a five-point Likert scale (0 = “not at all” to 4 = “very strongly”). Positively worded items in the Dental Self-Confidence domain were reverse-coded to align scoring direction [10].

Psychometric validation

To assess construct validity, an exploratory factor analysis (EFA) was conducted employing principal component extraction alongside varimax rotation. The data’s suitability for factor analysis was confirmed by achieving a Kaiser-Meyer-Olkin (KMO) measure of 0.80 or higher, and a highly significant result from Bartlett’s test of sphericity (p < 0.001). Factor loadings were considered meaningful if they exceeded 0.40 [10].

Convergent validity was evaluated using Spearman’s correlation between IOTN-AC scores and PIDAQ scores. Discriminant validity was evaluated using one-way ANOVA and post hoc comparisons to detect score differences across IOTN-based treatment need groups [10].

Statistical analysis

IBM SPSS Statistics, Version 21.0 (IBM Corp., Armonk, NY) was employed to perform the statistical analysis for this study. Descriptive statistics summarized participant characteristics, including means, standard deviations, and frequency distributions. Reliability was measured via Cronbach’s alpha, with values ≥0.70 indicating acceptable internal consistency, and test-retest reliability was assessed using two-way mixed model intraclass correlation coefficients (ICCs), with values ≥0.75 considered satisfactory. Validity assessments included EFA using principal component extraction and varimax rotation to evaluate construct validity. The suitability of data for EFA was confirmed through the Kaiser-Meyer-Olkin (KMO) measure and Bartlett’s test of sphericity. Factor loadings ≥0.40 were deemed meaningful. Spearman’s rank correlation coefficients were used to assess convergent validity by evaluating associations between PIDAQ scores and IOTN-AC grades. Discriminant validity was analyzed using one-way ANOVA to compare PIDAQ scores across malocclusion severity categories, followed by post hoc Tukey’s honestly significant difference (HSD) tests for intergroup comparisons. Statistical significance was determined at a threshold of p < 0.05.

## Results

A total of 450 adolescents aged 10 to 13 years participated in the study, with a mean age of 11.6 ± 1.1 years. The sample comprised 240 male and 210 female participants, primarily from urban and semi-urban areas. Table 1 summarizes the sociodemographic characteristics and corresponding PIDAQ scores. Female participants and those from urban regions exhibited higher mean PIDAQ scores, suggesting greater psychosocial sensitivity to dental aesthetics in these subgroups.

**Table 1:** Distribution of sociodemographic characteristics and orthodontic treatment needs among study participants. This table summarizes the distribution of gender and orthodontic treatment needs in a sample of 450 adolescents aged 10–13 years. Treatment needs were assessed using the Dental Health Component (DHC) and Aesthetic Component (AC) of the Index of Orthodontic Treatment Need (IOTN), categorized into three clinical grades: little, moderate, and definite treatment need.

Demographics	n (%)
Gender	
Male	223 (49.6)
Female	227 (50.4)
IOTN-DHC grades	
Little need for treatment (grades 1 and 2)	157 (34.9)
Moderate need (grade 3)	101 (22.4)
Definite need (grades 4 and 5)	192 (42.7)
IOTN-AC grades	
Little need for treatment (grades 1 – 4)	378 (84.0)
Moderate need (grades 5 – 7)	43 (9.6)
Definite need (grades 8 – 10)	29 (6.4)

Internal consistency of the Odia version of PIDAQ was confirmed by Cronbach’s alpha values ranging from 0.720 to 0.781 across the four domains. The highest reliability was observed in the DSC domain (α = 0.781), followed by SI, PI, and ACn, all exceeding the accepted threshold of 0.70. Test-retest reliability, assessed through intraclass correlation coefficients (ICC), ranged from 0.71 to 0.84. The DSC domain showed the highest ICC (0.84), followed by PI (0.81), SI (0.74), and ACn (0.71), indicating strong temporal stability. These findings are presented in Table 2.

**Table 2:** Item-wise factor loading after principal component analysis and varimax rotation with Kaiser normalization, amount and percentages of explained variance, Cronbach’s alpha if item deleted, and reliability of each subscale. * Salient factor loading. The values indicate the items showing similar factor loading under a particular domain. Factor structure of the Psychosocial Impact of Dental Aesthetics Questionnaire (PIDAQ) was derived using principal component analysis with varimax rotation. Items clustered under four domains: Social Impact (SI), Dental Self-Confidence (DSC), Psychological Impact (PI), and Aesthetic Concern (ACn). Cronbach’s alpha coefficients, both overall and for each subscale, along with variance explained, are presented to assess the psychometric properties and internal consistency of the instrument.

Items	Components				Cronbach’s alpha
	SI	DSC	PI	ACn	
Proud of teeth	0.068	0.800*	0.868	0.032	0.945
Like to show teeth	0.005	0.330*	0.051	0.996	0.812
Pleased to see teeth in the mirror	0.100	0.802*	0.52	-0.045	0.752
Teeth are attractive	-0.024	0.605*	0.023	-0.039	0.719
Satisfied with appearance	-0.152	0.421*	-0.047	0.156	0.769
Find tooth position nice	0.069	0.700*	-0.084	-0.012	0.834
Hold back when I smile	0.748*	0.107	0.099	0.090	0.967
What others think	0.821*	0.055	-0.074	0.045	0.926
Offensive remarks	0.800*	0.098	-0.042	0.125	0.816
Inhibited in social contacts	0.701*	-0.030	0.065	0.031	0.911
Hide my teeth	0.781*	-0.021	-0.095	0.737	0.927
People stare	0.892*	-0.139	0.046	0.020	0.795
Irritated on remarks	0.827*	0.018	-0.051	-0.043	0.813
Worry about opposite sex	0.745*	0.014	-0.087	0.009	0.791
Envy	0.102	-0.022	0.657*	0.900*	0.724
Somewhat distressed	0.011	0.116	0.721*	0.066	0.799
Somewhat unhappy	-0.008	-0.324	0.740*	0.287	0.759
Others have nicer teeth	-0.083	0.216	0.894*	0.253	0.981
Feel bad	0.729*	0.020	-0.087	-0.060	0.994
Wish teeth looked better	0.387	-0.197	0.531*	-0.415	0.839
Don’t like teeth in mirror	0.195	-0.001	0.033	0.641*	0.895
Don’t like teeth in photo	-0.038	0.001	0.018	0.830*	0.741
Don’t like teeth on video	0.047	0.002	-0.056	0.826*	0.701
Amount of variance explained (initial solution)	4.769	6.024	1.484	1.324	-
Percentage of variance explained (initial solution)	36.14	10.28	5.190	4.956	-
Percentage of variance explained (rotated solution)	20.84	15.12	10.52	9.467	-
Cronbach’s α	0.720	0.777	0.729	0.781	-

Construct validity was supported through EFA using principal component analysis with varimax rotation. A stable four-factor structure emerged, corresponding to SI, DSC, PI, and ACn. This structure accounted for 56.56% of the total variance, with all items demonstrating acceptable factor loadings above 0.4. The highest loading (0.892) was observed for the item “People stare” under the SI domain (Table 2).

The SI domain accounted for 20.84% of the total variance, followed by DSC at 15.12%, PI at 10.52%, and ACn at 9.46%. The distribution of variance across domains is detailed in Table 2.

Discriminant validity was established through significant differences in PIDAQ scores across malocclusion severity categories assessed by IOTN-DHC and IOTN-AC indices. Participants categorized as having definite orthodontic treatment need (grades 4-5) reported the highest psychosocial impact (mean total PIDAQ = 72.48 ± 3.86), followed by those with moderate (66.26 ± 4.08) and little or no treatment need (57.99 ± 3.56). One-way ANOVA indicated statistically significant differences across groups (p < 0.001). Domain-wise scores also increased with malocclusion severity, particularly in the SI and PI domains. These findings are summarized in Table 3.

**Table 3:** Comparison of scores among the DHC of the IOTN categorized groups using one-way ANOVA with post hoc Tukey test. Mean total PIDAQ scores across groups stratified by DHC-based treatment need (little, moderate, and definite) were compared using one-way analysis of variance. Post hoc Tukey tests evaluated pairwise differences, with statistically significant intergroup differences supporting the discriminant validity of the instrument. PIDAC: Psychosocial Impact of Dental Aesthetics Questionnaire, IOTN: Index of Orthodontic Treatment Need, DHC: Dental Health Component, SE: Standard Error.

One-way ANOVA				Post hoc Tukey test			
IOTN-DHC categorized groups	N	PIDAQ score mean (SE)	p-value	Intergroup comparison	Difference in PIDAQ score, mean (SE)	p-value	95% Confidence Interval
Little need of treatment	157	57.99 (3.564)	0.000	Little need – moderate need	-8.27 (2.561)	0.002	-14.21, -2.94
Moderate need	101	66.26 (4.083)		Moderate need – definite need	6.22 (2.964)	0.191	-15.46, 1.85
Definite need	192	72.48 (3.860)		Little need – definite need	-14.99 (3.528)	0.000	-22.51, -8.01
Total	450	65.89 (3.635)					

Convergent validity was demonstrated by statistically significant positive correlations between PIDAQ scores and IOTN-AC scores. The total PIDAQ score correlated at ρ = 0.389 (p < 0.05), with the strongest domain-level correlation in DSC (ρ = 0.356), followed by PI (ρ = 0.314), SI (ρ = 0.257), and ACn (ρ = 0.201). These results confirm that self-perceived dental aesthetics are associated with psychosocial impact, supporting the scale’s convergent validity. Full correlation values are presented in Table 4.

**Table 4:** Comparison and correlation of the domain and total scores in subjects with different self-rated ACs of the IOTN scores using one-way ANOVA. Mean scores across the four PIDAQ domains and the total score were compared among groups stratified by self-rated aesthetic need (IOTN-AC grades 1–4, 5–7, and 8–10) using one-way ANOVA. Additionally, Spearman’s correlation coefficients quantified the association between self-perceived aesthetic concern and psychosocial impact, thereby demonstrating the convergent validity of the PIDAQ. PIDAQ: Psychosocial Impact of Dental Aesthetics Questionnaire, IOTN: Index of Orthodontic Treatment Need, AC: Aesthetic Component, SE: Standard Error, F: statistics and level of significance.

Domain/score	Mean (SE) 1–4 (n=378)	Mean (SE) 5–7 (n=43)	Mean (SE) 8–10 (n=29)	F-statistic (p-value)	Spearman’s ρ (p-value)
Dental Self-Confidence	20.00 (0.421)	24.56 (0.947)	22.49 (1.584)	9.264 (p < 0.001)	0.356 (p < 0.05)
Social Impact	18.01 (0.524)	22.53 (1.654)	21.62 (1.899)	5.961 (p < 0.001)	0.257 (p < 0.05)
Psychological Impact	17.09 (6.012)	19.51 (1.789)	19.28 (1.368)	4.789 (p < 0.001)	0.314 (p < 0.05)
Aesthetic Concern	6.578 (0.851)	8.023 (0.915)	8.942 (0.858)	5.128 (p < 0.001)	0.201 (p < 0.05)
PIDAQ Total	61.67 (1.564)	74.62 (3.955)	72.03 (4.125)	9.123 (p < 0.001)	0.389 (p < 0.05)

## Discussion

This research marks the initial attempt to linguistically translate, culturally tailor, and evaluate the psychometric properties of PIDAQ for use in the Odia-speaking population, addressing a crucial gap in orthodontic assessment tools for Eastern Indian adolescents. Adolescence is a formative period during which self-concept and appearance become tightly linked with psychological well-being, peer acceptance, and social participation [2]. Dental aesthetics, particularly malocclusion, can significantly influence this developmental trajectory. While the PIDAQ has been validated in several global and Indian contexts-including Spanish [3], Moroccan [4], Chinese [5], Turkish [6], Persian [7], Hindi [8,9], and Malayalam [10] before this study, no such adaptation had been available for Odia-speaking populations, despite Odisha being home to over 35 million Odia speakers [16].

The PIDAQ is a widely recognized psychometric tool developed to assess the subjective, psychosocial effects of dental aesthetics on individuals [1,2]. It comprises four distinct domains (DSC, SI, PI, and ACn), making it a comprehensive instrument not just for clinical research but also for public health planning and policy development [1,2]. The cultural sensitivity of PIDAQ to local perceptions of aesthetics and self-esteem makes linguistic and contextual adaptation vital for its valid application across diverse populations [4,10,13].

This study addressed that gap by translating and adapting the PIDAQ following internationally accepted procedures for cross-cultural adaptation, as outlined by Herdman et al. [19]. These include forward translation by bilingual experts, synthesis by a multidisciplinary committee, backward translation, and pilot testing for semantic, idiomatic, and conceptual equivalence. Such methodology has also been followed in validations across Croatian [12], Malaysian [13], Persian [7], and Chinese [5] populations. The Odia version demonstrated high comprehension, clear layout, and average completion time (13.5 minutes) comparable to the Spanish (14 minutes) [3], Portuguese (13 minutes) [20], and Malaysian (12-15 minutes) [13] adaptations, confirming its operational equivalence.

A total of 450 adolescents aged 10 to 13 years participated in the study. The sample size was chosen based on Plichta and Kelvin’s guideline of 10 participants per item for psychometric validation (23 items × 10 = 230), with an additional buffer to accommodate potential attrition and to allow robust subgroup analyses [18]. This sampling strategy aligns with other large validation studies in Malaysian [13], Australian [21], and Chinese [5] contexts. Moreover, the selected age group aligns with prior validations, as adolescents in this developmental window exhibit heightened awareness of physical appearance, making them particularly sensitive to the psychosocial effects of dental aesthetics [2,3,8,10,14].

The psychometric evaluation of the Odia version of the PIDAQ demonstrated high internal consistency, evidenced by Cronbach’s alpha coefficients between 0.720 and 0.781 across its four subscales. These values exceed the standard threshold of 0.70, indicating good internal reliability [10]. However, they are slightly lower than those reported in more culturally homogenous populations, such as Malayalam (0.83-0.88) [10], Persian (0.809-0.886) [7], Hindi (0.86-0.92) [8], and Portuguese (0.93-0.98) [20] validations. The Turkish version showed more variability, with alpha values ranging from 0.534 to 0.904 [6], likely reflecting similar linguistic diversity challenges faced in Odisha.

Test and retest reliability, assessed via ICC, ranged from 0.71 to 0.84, confirming the Odia version’s temporal stability. These results are comparable to the ICCs in Hindi (0.84-0.92) [8,9], Persian (0.82-0.90) [7], Chinese (0.84-0.93) [5], and Arabic (0.937) [11] validations. High reproducibility across time points is critical for longitudinal clinical or epidemiological studies that aim to track psychosocial changes with orthodontic treatment.

Construct validity was established through exploratory factor analysis (EFA), which confirmed a stable four-factor structure accounting for 56.56% of the total variance-consistent with the original German model (63.6%) [1] and other cross-cultural adaptations, such as Moroccan (59.1%) [4], Chinese (58.9%) [5], Hindi (58.3%) [8], and Croatian (58.7%) [12]. Slightly lower explained variance in the Odia context could stem from sociolinguistic diversity and wider variance in self-perception among Indian adolescents.

At the domain level, the variance explained by SI (20.84%), DSC (15.12%), PI (10.52%), and ACn (9.46%) mirrors those seen in Hindi [8,9], Moroccan [4], Turkish [6], Persian [7], Portuguese [20], Swedish [22], and Malaysian [13] adaptations. For example, the Portuguese study reported domain variances of SI (23.2%), DSC (18.1%), and ACn (11.1%) [20], while the Swedish adolescent version showed SI = 22.7% and DSC = 17.4% [22]. These findings reinforce the structural fidelity of the PIDAQ across diverse populations, including Eastern India.

Discriminant validity was demonstrated through significant differences in PIDAQ scores across malocclusion severity levels, categorized using IOTN-DHC and IOTN-AC. Participants with definite need for treatment (DHC grades 4-5) reported significantly higher psychosocial impact (mean = 72.48 ± 3.86), followed by moderate need (66.26 ± 4.08) and little to no need (57.99 ± 3.56). This gradient mirrors findings from validations in Malayalam [10], Hindi [8,9], Chinese [5], and Australian [21] samples. ANOVA with post hoc testing confirmed the statistical significance of these differences, affirming the scale’s clinical sensitivity in detecting psychosocial burden corresponding to increasing malocclusion severity.

Convergent validity was confirmed by a statistically significant positive correlation between IOTN-AC scores and total PIDAQ scores (ρ = 0.389, p < 0.05), consistent with findings from the Persian [7], Moroccan [4], and Spanish [3] versions. Among subdomains, DSC (ρ = 0.356) showed the strongest correlation, followed by PI (ρ = 0.314), SI (ρ = 0.257), and ACn (ρ = 0.201), closely aligning with Malayalam (0.41-0.47) [10] and Hindi (≈0.42) [8,9] findings. These results suggest that self-perception of dental aesthetics aligns with measurable psychosocial outcomes, reinforcing the validity of PIDAQ as a patient-reported outcome tool.

The study’s findings confirmed the primary hypothesis by establishing the reliability and validity of the Odia-adapted PIDAQ. Additionally, the secondary null hypothesis was rejected, as results demonstrated a significant association between malocclusion severity and the psychosocial impact of dental aesthetics among Odia-speaking adolescents. These findings are consistent with previous Indian validations of the PIDAQ in Hindi [8,9] and Malayalam [10], which also reported significant psychosocial impact associated with increasing malocclusion severity, thereby reinforcing the clinical sensitivity and cultural adaptability of the instrument across diverse Indian populations.

While the study’s strengths lie in its robust psychometric methodology, cultural sensitivity, and large sample size, certain limitations warrant consideration. The participant pool was limited to school-going adolescents aged 10 to 13 years from urban and semi-urban settings, potentially excluding perspectives from rural and tribal populations with differing aesthetic norms and health literacy. As a result, generalizability to older adolescents or rural demographics may be limited. Additionally, while the cross-cultural adaptation process followed established standards [22], subtle cultural interpretations of certain items may still vary. The study also did not evaluate predictive validity or responsiveness over time-parameters essential for assessing treatment-related psychosocial changes. Furthermore, the use of self-reported data raises the possibility of social desirability bias, particularly in domains such as self-confidence and psychological impact.

Future research should aim to address these limitations by incorporating rural and tribal samples, conducting longitudinal studies to assess responsiveness to orthodontic interventions, and exploring predictive validity. Qualitative methods may also help uncover cultural constructs related to dental appearance and self-esteem. Given its psychometric robustness, the validated Odia PIDAQ holds substantial potential for use in large-scale oral health surveillance, school screening programs, and public health planning. Its application can help clinicians and policymakers identify adolescents at risk of psychosocial distress related to dental aesthetics, thus enabling timely and holistic interventions.

## Conclusions

The present study successfully bridges a significant cultural and linguistic gap in orthodontic psychosocial assessment by developing and validating the Odia version of the PIDAQ for adolescents. Through rigorous cross-cultural adaptation and psychometric validation, the instrument demonstrated good reliability, a stable four-domain factor structure, and strong construct, discriminant, and convergent validity, confirming its appropriateness for capturing the nuanced psychosocial impact of dental aesthetics in Eastern Indian adolescents. The findings revealed a clear, progressive relationship between the severity of malocclusion and psychosocial burden, with heightened sensitivity noted among female and urban participants. By correlating clinical indices like IOTN-DHC and IOTN-AC with PIDAQ scores, the study underscores the importance of integrating subjective self-perception with objective clinical assessments in orthodontic diagnosis and treatment planning. Importantly, this research not only validates a culturally sensitive tool for clinical and public health use but also highlights the potential of patient-reported outcomes in identifying adolescents at psychosocial risk due to dental aesthetic concerns. The Odia PIDAQ thus emerges as a pivotal resource for clinicians, educators, and policymakers striving for comprehensive, patient-centered orthodontic care in a linguistically diverse population.

## Appendices

**Table 5:** Modified Psychosocial Impact of Dental Aesthetics Questionnaire (PIDAQ) (Odia) and original PIDAQ. Credits to modified PIDAQ (Odia): Vijeta Patri, Gaurav Patri, Nivedita Sahoo. Translated and reproduced by permission of Oxford University Press. Oxford University Press is not responsible for the accuracy of the translation. Original PIDAQ reproduced from Klages et al., 2006 [1] by permission of Oxford University Press and Copyright Clearance Center (License Number 6001731188461).

Original PIDAQ	Modified PIDAQ in Odia
Dental Self-Confidence (DSC)	ଦାନ୍ତରେ ଆତ୍ମବିଶ୍ୱାସ
I am proud of my teeth.	ମୁଁ ମୋର ଦାନ୍ତ ପାଇଁ ଗର୍ବିତ |
I like to show my teeth when I smile.	ମୁଁ ହସିବାବେଳେ ମୋର ଦାନ୍ତ ଦେଖାଇବାକୁ ଭଲପାଏ |
I am pleased when I see my teeth in the mirror.	ମୁଁ ଦର୍ପଣରେ ମୋର ଦାନ୍ତ ଦେଖି ଖୁସି ହୁଏ |
My teeth are attractive to others.	ମୋର ଦାନ୍ତ ଅନ୍ୟମାନଙ୍କ ପାଇଁ ଆକର୍ଷଣୀଯ଼ |
I am satisfied with the appearance of my teeth.	ମୁଁ ମୋ ଦାନ୍ତର ରୂପକୁ ନେଇ ସନ୍ତୁଷ୍ଟ।
I find my tooth position to be very nice.	ମୋର ଦାନ୍ତର ସ୍ଥିତି ବହୁତ ଭଲ ଲାଗୁଛି।
Social Impact (SI)	ସାମାଜିକ ପ୍ରଭାବ
I hold myself back when I smile so my teeth don’t show so much.	ଯେତେବେଳେ ମୁଁ ହସୁଥାଏ, ମୁଁ ନିଜକୁ ଅଟକାଇ ରଖିଥାଏ, ଯାହାଦ୍ୱାରା ମୋର ଦାନ୍ତ ଏତେ ଅଧିକ ଦେଖାଯିବ ନାହିଁ। |
If I don’t know people well, I am sometimes concerned about what they might think about my teeth.	ଯଦି ମୁଁ ଲୋକମାନଙ୍କୁ ଭଲ ଭାବରେ ଜାଣେ ନାହିଁ, ମୁଁ ବେଳେବେଳେ ଚିନ୍ତିତ ଯେ ସେମାନେ ମୋ ଦାନ୍ତ ବିଷଯ଼ରେ କ 'ଣ ଭାବିବେ।
I’m afraid that other people could make offensive remarks about my teeth.	ମୁଁ ଭଯ଼ କରୁଛି ଯେ ଅନ୍ଯ଼ମାନେ ମୋ ଦାନ୍ତ ବିଷଯ଼ରେ ଆପତ୍ତିଜନକ ମନ୍ତବ୍ଯ଼ ଦେଇପାରନ୍ତି।
I am somewhat inhibited in social situations, because of my teeth.	ମୋର ଦାନ୍ତ କାରଣରୁ ମୁଁ ସାମାଜିକ ଯୋଗାଯୋଗରେ କିଛି ମାତ୍ରାରେ ବାଧାପ୍ରାପ୍ତ।
I sometimes catch myself holding my hand in front of my mouth to hide my teeth.	ବେଳେବେଳେ ମୁଁ ଦାନ୍ତ ଲୁଚାଇବା ପାଇଁ ମୋ ମୁହଁ ସାମ୍ନାରେ ହାତ ଧରିଥାଏ।
Sometimes, I think people are staring at my teeth.	ବେଳେବେଳେ ମୁଁ ଭାବୁଛି ଲୋକମାନେ ମୋ ଦାନ୍ତକୁ ଚାହିଁ ବସିଛନ୍ତି।
Remarks about my teeth irritate me, even when they are meant jokingly.	ମୋ ଦାନ୍ତ ବିଷଯ଼ରେ ମନ୍ତବ୍ଯ଼ ମୋତେ ବିରକ୍ତ କରେ ଯଦିଓ ସେଗୁଡ଼ିକ ମଜାଳିଆ ଭାବରେ ବୁଝାଏ
I sometimes worry about what members of the opposite sex think about my teeth.	ମୁଁ ବେଳେବେଳେ ଚିନ୍ତା କରେ ଯେ ବିପରୀତ ଲିଙ୍ଗର ସଦସ୍ଯ଼ମାନେ ମୋ ଦାନ୍ତ ବିଷଯ଼ରେ କ 'ଣ ଭାବନ୍ତି।
Psychological Impact (PI)	ମାନସିକ ପ୍ରଭାବ
I envy the nice teeth of other people.	ମୁଁ ଅନ୍ୟ ଲୋକଙ୍କ ସୁନ୍ଦର ଦାନ୍ତକୁ ଈର୍ଷା କରେ |
I am somewhat distressed when I see other people’s teeth.	ଯେତେବେଳେ ମୁଁ ଅନ୍ଯ଼ ଲୋକଙ୍କ ଦାନ୍ତ ଦେଖେ, ମୁଁ ଟିକେ ବିଚଳିତ ହୁଏ।.
Sometimes, I am somewhat unhappy about the appearance of my teeth.	ବେଳେବେଳେ ମୁଁ ମୋର ଦାନ୍ତର ରୂପକୁ ନେଇ କିଛିଟା ଅସନ୍ତୁଷ୍ଟ।
I think that most people I know have nicer teeth than I do.	ମୁଁ ଭାବୁଛି ମୁଁ ଜାଣିଥିବା ଅଧିକାଂଶ ଲୋକଙ୍କର ମୋ ଠାରୁ ଭଲ ଦାନ୍ତ ଅଛି।
I feel bad when I think about what my teeth look like.	ଯେତେବେଳେ ମୁଁ ମୋ ଦାନ୍ତ କିପରି ଦେଖାଯାଏ ସେ ବିଷଯ଼ରେ ଚିନ୍ତା କରେ ମୁଁ ଖରାପ ଅନୁଭବ କରେ |
I wish my teeth looked better.	ମୁଁ ଚାହେଁ ମୋ ଦାନ୍ତ ଭଲ ଦେଖାଯାଉ।
Aesthetic Concern (ACn)	ସୌନ୍ଦର୍ଯ୍ଯ଼ ସମ୍ବନ୍ଧୀଯ଼ ଚିନ୍ତା
I don’t like to see my teeth in the mirror.	ମୁଁ ଦର୍ପଣରେ ମୋର ଦାନ୍ତ ଦେଖିବାକୁ ପସନ୍ଦ କରେ ନାହିଁ।
I don’t like to see my teeth in photographs.	ଫଟୋଗୁଡ଼ିକରେ ମୋର ଦାନ୍ତ ଦେଖିବାକୁ ମୁଁ ପସନ୍ଦ କରେ ନାହିଁ।
I don’t like to see my teeth when I look at a video of myself.	ଯେତେବେଳେ ମୁଁ ନିଜର ଏକ ଭିଡିଓ ଦେଖେ, ମୁଁ ମୋର ଦାନ୍ତ ଦେଖିବାକୁ ପସନ୍ଦ କରେ ନାହିଁ।
Responses	
0 – not at all	ଆଦୌ ନୁହେଁ
1 – a liitle	ଅଳ୍ପ
2 – somewhat	କିଛି ମାତ୍ରାରେ
3 – strongly	ଦୃଢ଼ ଭାବେ
4 – very strongly	ଅତି ଦୃଢ଼ ଭାବେ
